# Model of Metal Microstructure Evolution Considering Shear Effect and Its Simulation Application in Rolling of Heavy Cylinders

**DOI:** 10.3390/ma14061500

**Published:** 2021-03-18

**Authors:** Yunjing Jiao, Zhikui Dong, Pengwei Liang, Jianliang Sun

**Affiliations:** 1 School of Mechanical Engineering, Yanshan University, Qinhuangdao 066004, Hebei, China; jyj18830970939@126.com (Y.J.); lpwfting@126.com (P.L.); sunjianliang@ysu.edu.cn (J.S.); 2 National Engineering Research Center for Equipment and Technology of C.S.R., Yanshan University, Qinhuangdao 066004, Hebei, China

**Keywords:** shear effect, heavy cylinder, secondary development program, microstructure evolution

## Abstract

In the rolling process of heavy cylinders, the deformation section is subjected to the effects of compression and shear. In order to analyze the influences of the shear effect on the microstructure evolution characteristics, a mathematical model was established and the rolling process was simulated. Firstly, shear-compression specimen (SCS) and ordinary cylinder specimens were designed, high-temperature compression experiments were carried out and the mathematical model of microstructure evolution considering shear effect was established; then, a program based on finite element software was developed to simulate the microstructure evolution process, and the feasibility of the development program was verified by compression experiments. Finally, a macro–micro coupling model based on the development program was established to simulate the microstructure evolution of the heavy cylinder during the rolling process. Then, the influence of the shear effect on the microstructure evolution was analyzed. The results showed that the shear effect had a great influence on the heavy cylinder. Dynamic recrystallization was more likely to occur in the heavy cylinder during the rolling process and the grain refinement was more obvious; compared with the case without considering the shear effect, the volume fraction of dynamic recrystallization was increased by 0.25%, and the grain size was refined by 30 μm.

## 1. Introduction

The heavy cylinder is the basic component of heavy pressure vessels, which are widely used in nuclear power and the petrochemical, coal liquefaction and aerospace industries [1]. The size of heavy cylinders is large: the diameter of these cylinders can reach 15 m, the height is about 3 m the wall thickness is more than 0.3 m [2]. In the rolling process of heavy cylinders, the microstructure is also changed alongside the macroscopic deformation. The change of the microstructure ultimately determines the forming quality of the heavy cylinder. Therefore, it is of great significance to study the microstructure evolution of the metal material. At present, the research into the microstructure of metal materials has mainly adopted the method of the simple physical analogue and finite element simulation, in which the physical analogue mainly focuses on the compression behavior. However, there is a special section, the compression-shear section, that is affected by the compression shear effect in the rolling process of heavy cylinders [3], and thus a simple compression experiment is not sufficient to study the influence of the shear effect. In addition, the shear effect is not considered in the current finite element simulation. Therefore, it is necessary to analyze the shear effect and simulate the microstructure evolution process of heavy cylinders during the rolling process.

During the hot deformation of metal, the internal structure of the material changes from coarse grains to finer grains with a more compact structure due to the effect of the external force. Meanwhile, the metal undergoes different changes caused by the distinct deformation temperature, strain rate and strain, mainly including dynamic recovery, recrystallization and work hardening. Many studies have been performed investigating the aspects of the theory and have conducted experiments on the microstructure, but they have mainly focused on the establishment of a recrystallization model rather than the simulation of the microstructure evolution. For example, Qin and Li [4] studied the effect of heating specification on the grain growth of low-carbon steel and established the grain growth model; furthermore, they analyzed the microstructure and mechanical properties of the rolled ring (a small-sized ring), and their evolution laws during quenching and tempering were discussed [5]. The authors provided a reference for a method to study the grain structure of 2.25Cr1Mo0.25V steel and the microstructure of heavy cylinders under different deformation conditions. In addition, Dai et al. [6] studied the microstructure evolution of 5083 aluminum alloys and established a relevant grain mathematical model. Sun et al. [7] established the dynamic recrystallization model of a 2A14 aluminum alloy by an isothermal compression experiment. However, the above studies were all based on a single compression analogue, and the shear effect was not included in the microstructure evolution model. Now, the research on the shear effect is mainly applied to the shear deformation of geotechnical materials, including the effect of compressive-shear loading on roller compacted concrete [8] and compressive-shear cracks on granites [9]. The research into metal has been relatively scarce, mainly using the Hopkinson compression shear bar and designing experimental specimens. Rittel et al. [10] designed a shear-compression specimen (SCS) to realize the shear effect and proved by finite element simulation that the specimen could achieve a higher strain rate and strain at room temperature [11]; Sang et al. [12] studied the compression shear deformation of a metal at high temperature, but the materials selected were difficult to use as a deformation material with a single size and were thus not suitable for this paper.

A microstructural simulation can directly monitor the microstructure evolution of the product forming process and guide the manufacturing process [13,14,15]. Wang et al. [13] compiled a subroutine of microstructure evolution to simulate the microstructure evolution of the β phase in a titanium alloy ring. Li et al. [14] analyzed the research status of microstructures in the ring rolling process and showed a direction for further research. Ji et al. studied the dynamic recrystallization behavior and microstructure evolution of 33Cr23Ni8Mn3N heat-resistant steel, then imported the established model into Deform-3D, and hot compression was simulated by the finite element method [15]. The above studies provide an important illustration, but the microstructure prediction of the whole process of product formation could not be realized, and only one aspect was considered, thus representing incomplete research. Thus, in this paper, the microstructure evolution model of the metal material deformation process was established based on the shear effect, and through the secondary development of the finite element software, simulation research on the microstructure evolution of the cylinder rolling process was realized.

In the current study, the process of the microstructure evolution of heavy cylinder rolling was studied. This paper is organized in five sections. The first section introduces the subject of this work. The second section presents the high-temperature compression experiment of the SCS and ordinary cylinder specimen and then analyzes the influence of the shear effect. The third section presents the mathematical model of dynamic recrystallization for the SCS and ordinary cylinder specimen. The fourth section presents the program of microstructure evolution, which is then verified. The macro–micro coupled model of heavy cylinder rolling is then described. The fifth section presents the microstructure evolution characteristics of the rolling process and demonstrates the importance of the shear effect.

## 2. Experiment Research

### 2.1. Experimental Scheme

The material of the test specimen was 2.25Cr1Mo0.25V steel, and its chemical composition is shown in Table 1.

**Figure 1:**
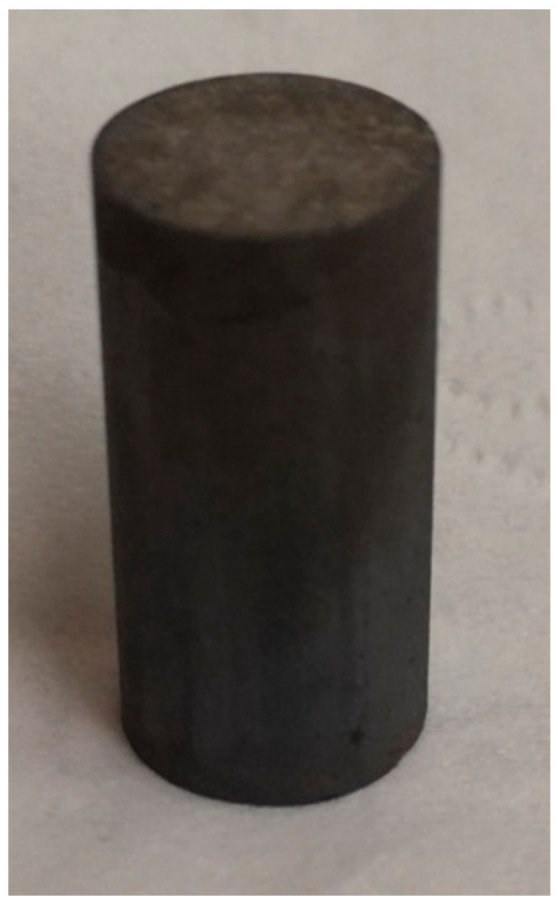
Sample of common cylindrical specimen.

**Figure 2:**
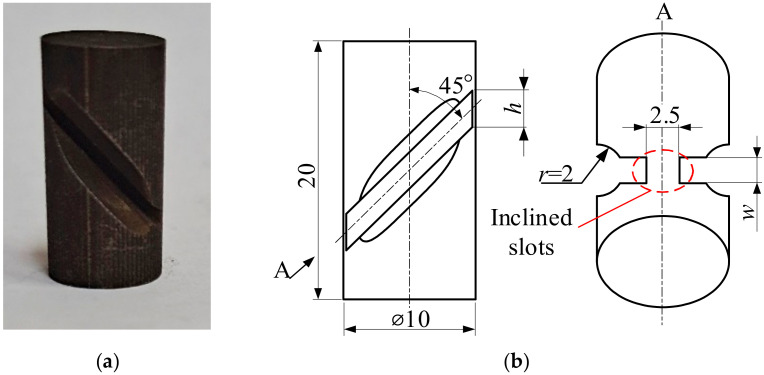
Sample of a shear-compression specimen (SCS). (**a**) Description of physical map; (**b**) description of size map (unit of measurement is mm).

A common cylindrical specimen is presented in Figure 1 and an SCS in Figure 2. The end faces only bore the pressure P. The specimens had dimensions of Ø10 × 20 mm, and the upper and lower circular end faces were finely turned. In the SCS, two inclined slots were machined along an angle of 45° to the longitudinal axis of the cylinder. There were expansion slots with *r* = 2 mm at the inclined slots to allow the shear zone to bear the combined action of compression and shear. The size of the shear zone was determined by the width (*w*) and thickness (*t*) of the slot. The thickness was *t* = 2.5 mm, and the width of the slot was *w* = 1.0 mm.

**Figure 3:**
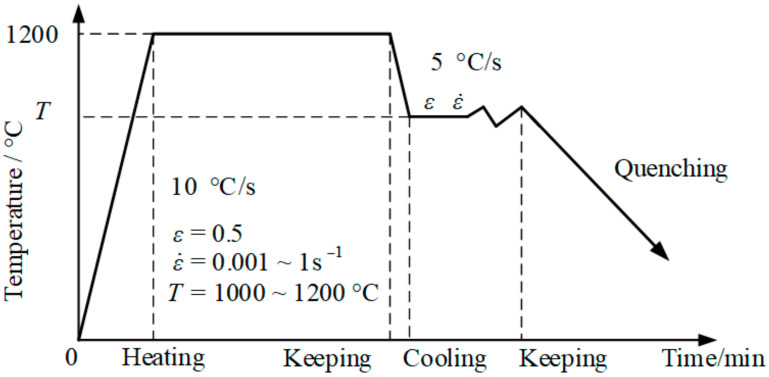
Experimental scheme.

**Figure 4:**
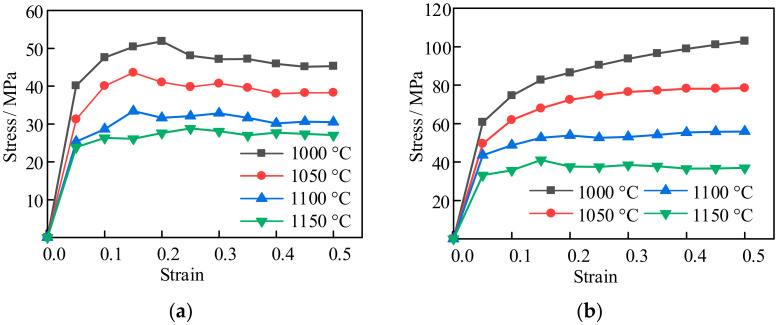

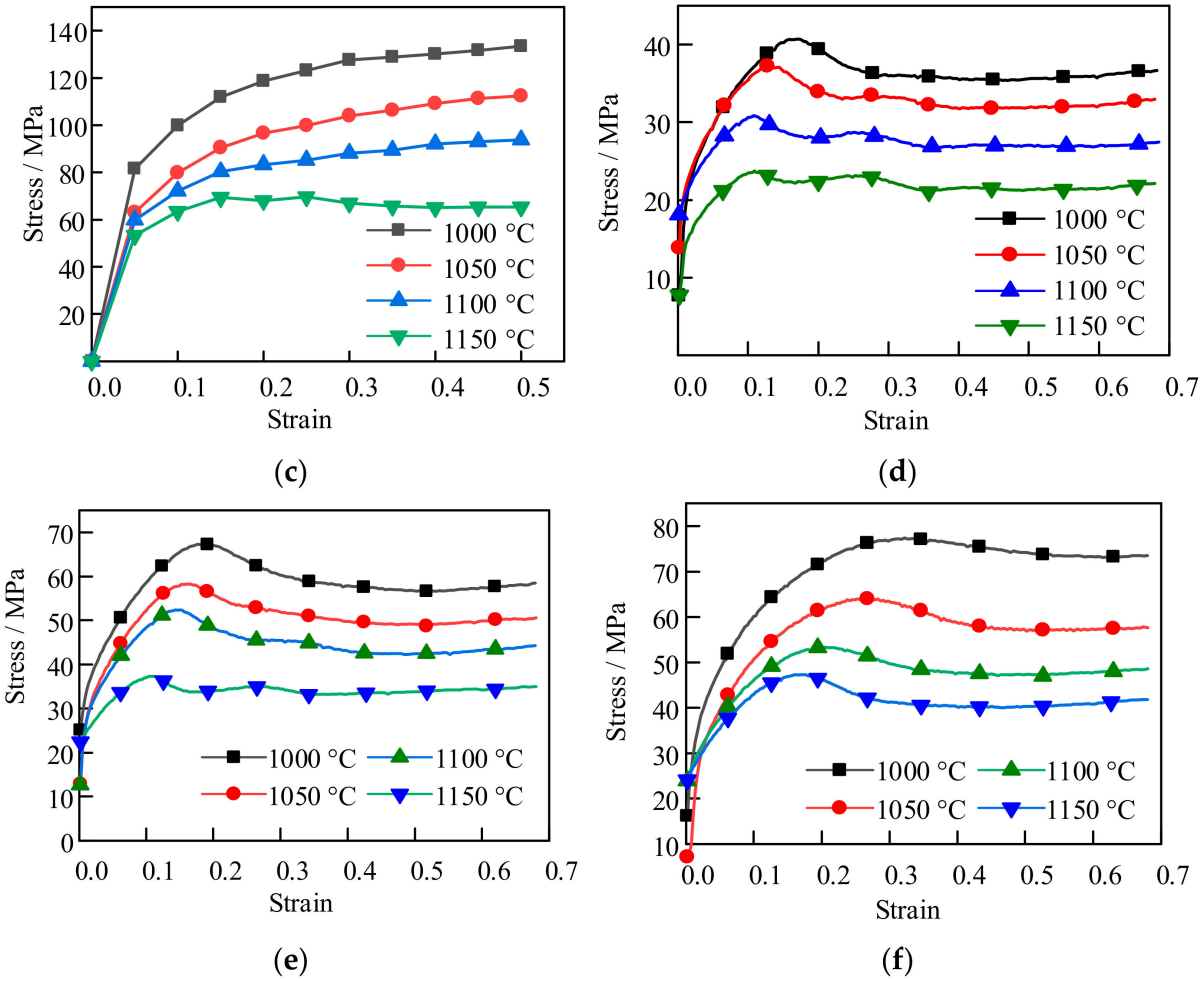
Sample of stress–strain curves: (**a**) common cylindrical specimen at $\overset{\cdot }{\varepsilon }$ = 0.001 s^−1^; (**b**) common cylindrical specimen at $\overset{\cdot }{\varepsilon }$ = 0.01 s^−1^; (**c**) common cylindrical specimen at $\overset{\cdot }{\varepsilon }$ = 0.1 s^−1^; (**d**) SCS at $\overset{\cdot }{\varepsilon }$ = 0.001 s^−1^; (**e**) SCS at $\overset{\cdot }{\varepsilon }$ = 0.01 s^−1^; (**f**) SCS at $\overset{\cdot }{\varepsilon }$ = 0.1 s^−1^.

The experimental scheme is shown in Figure 3. All specimens were heated to 1200 °C at the heating rate of 10 °C/s and kept for 5 min, then cooled to the strain temperature at the rate of 5 °C/s and kept for 2 min and finally compressed. The strain temperatures were 1000 °C, 1050 °C, 1100 °C, and 1150 °C, and the compression rates were 0.001 s^−1^, 0.01 s^−1^ and 0.1 s^−1^, respectively. The true strain was 0.5. Figure 4a shows the comparison results of the ordinary cylindrical specimens with a strain rate of 0.001 s^−1^ at different strain temperatures. The strain temperatures were set as mentioned above. Since the parameters collected by the Gleeble-3800 (Gleeble, New York, NY, USA) machine were automatically given by the computer according to the calculation equation of a cylinder, which was not suitable for the SCS, the relevant parameters were first processed, and the processing occurred according to the method presented in [16].

### 2.2. Analysis and Discussion of Experimental Results

The experimental results are shown in Figure 4, where Figure 4a–c show the s tress-strain curves of the common cylindrical specimen and Figure 4d–f show the stress-strain curves of the SCS. From the figures, dynamic recrystallization was observed under the experimental conditions shown in Figure 4a,d,e. The deformation conditions of Figure 4a,d were $\overset{\cdot }{\varepsilon }$ = 0.001 s^−1^, T ≥ 1000 °C, and Figure 4e was $\overset{\cdot }{\varepsilon }$ = 0.01 s^−1^, T ≥ 1000 °C. In Figure 4b, dynamic recrystallization occurred when the deformation condition was $\overset{\cdot }{\varepsilon }$ = 0.01 s^−1^, T ≥ 1100 °C. In Figure 4c, dynamic recrystallization occurred when the deformation condition was $\overset{\cdot }{\varepsilon }$ = 0.1 s^−1^, T ≥ 1150 °C. In Figure 4f, dynamic recrystallization occurred in all the deformation conditions. Dynamic recovery occurred in all other deformation conditions.

From Figure 4: In the process of hot deformation, the higher the deformation temperature, the smaller the deformation resistance when the strain rate remained constant; due to the existence of a shear band, the stress of SCS was less than that of the ordinary cylinder specimen under the same deformation condition.

It was found that the lower the deformation temperature, the larger the peak strain when the deformation rate was constant; i.e., the decrease in temperature inhibited the dynamic recrystallization.

On the premise that the deformation temperature and strain remained unchanged, the higher the strain rate, the greater the stress peak, and the corresponding peak strain was also larger; i.e., the increase in strain rate inhibited the occurrence of dynamic recrystallization.

Compared with the deformation curves of the cylinder specimen and SCS, it was found that dynamic recrystallization occurred more easily in SCS due to the existence of the shear band under the same deformation conditions.

In conclusion, dynamic recrystallization was more likely to occur in SCS under the same deformation conditions.

## 3. Establishment of Mathematical Model for Dynamic Recrystallization

### 3.1. Study of the Dynamic Recrystallization Kinetic Model

The dynamic recrystallization model of metal materials forms the basis for the further study of the materials’ properties, which have been studied by many researchers. A mature theoretical model [17] was adopted in this paper as follows:

(1) $$X_{d}=1-\exp\left[-\beta_{d}{\left(\frac{\varepsilon -\varepsilon_{c}}{\varepsilon_{p}}\right)}^{k_{d}}\right]$$

(2) $$\varepsilon_{p}=A_{1}Z^{m_{1}}$$

(3) $$\varepsilon_{c}=A_{2}Z^{m_{2}}$$

(4) $$Z=\overset{\cdot }{\varepsilon }\exp[Q_{d}/(RT)]$$

where *X_d_* is the dynamic recrystallization volume fraction; *K_d_* and *β_d_* are correlation coefficients; *ε* is the strain; $\overset{\cdot }{\varepsilon }$ is the strain rate; *ε_p_* is the peak strain; *ε_c_* is the critical strain; *A*_1_, *A*_2_, *m*_1_ and *m*_2_ are the material correlation coefficients; *Q_d_* is the dynamic recrystallization activation energy; *R* is the gas constant, *R* = 8.314 J/mol; and *T* is the temperature, °C.

The subscripts c and s were used to represent the common cylindrical specimen and the SCS, respectively, to distinguish the two kinds of specimens. The dynamic recrystallization kinetic models established for the two specimens were as follows.

Common cylindrical specimen:

(5) $$X_{dc}=1-\exp\left[-1.55671{\left(\frac{\varepsilon -\varepsilon_{c}}{\varepsilon_{p}}\right)}^{0.40822}\right]$$

(6) $$\varepsilon_{pc}=2.9250\times 10^{-3}Z_{c}^{0.24529}$$

(7) $$\varepsilon_{cc}=2.0346\times 10^{-4}Z_{c}^{0.30691}$$

(8) $$Z_{c}=\overset{\cdot }{\varepsilon }\exp[218628/(RT)]$$

Compression shear specimen:

(9) $$X_{ds}=1-\exp\left[-0.81108{\left(\frac{\varepsilon -\varepsilon_{c}}{\varepsilon_{p}}\right)}^{1.21193}\right]$$

(10) $$\varepsilon_{ps}=7.5810\times 10^{-3}Z_{s}^{0.13712}$$

(11) $$\varepsilon_{cs}=1.9438\times 10^{-3}Z_{s}^{0.15436}$$

(12) $$Z_{s}=\overset{\cdot }{\varepsilon }\exp[239657/(RT)]$$

### 3.2. Study of the Grain Size Model of Dynamic Recrystallization

**Figure 5:**
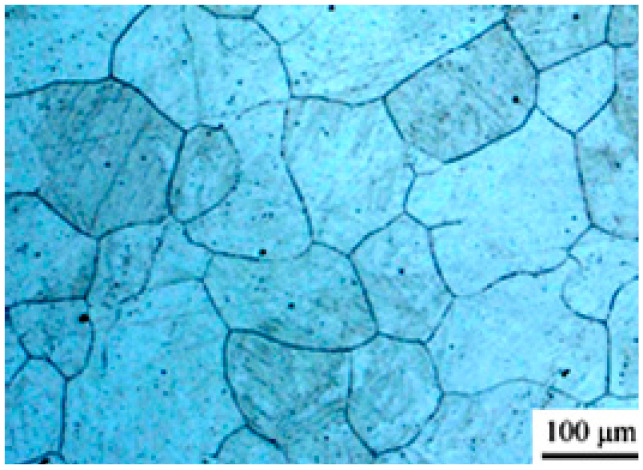
Initial grain size.

The dynamic recrystallization grain size model revealed the evolution characteristics of the grain size. It was impossible to carry out a metallographic structure observation experiment due to the limitations of time and the conditions. Therefore, the grain size was predicted based on previous research work [18] to lay the foundation for the following simulation work. (1) For the same specimen, the grain size increased with the increase in deformation temperature, when the deformation rate remained constant; (2) for the same specimen, the grain size increased with the decrease in the deformation rate, when the deformation temperature remained constant; (3) compared with the common cylinder specimen and the SCS, the grain size of the SCS was smaller and the refinement effect of dynamic recrystallization was better under the same deformation conditions. The initial grain size of the 2.25Cr1Mo0.25V was 129 μm, as shown in Figure 5. The predicted data are shown in Table 2.

The grain size *d_d_* of dynamic recrystallization was related to the deformation temperature *T* and the strain rate $\overset{\cdot }{\varepsilon }$, which could be expressed by the following formula [18]:

(13) $$d_{d}=G_{d}Z^{m_{d}}$$

where *G_d_* and *m_d_* refer to the dynamic recrystallization volume fraction.

The dynamic recrystallization grain size models of the two specimens were established as follows:

Common cylindrical specimen:

(14) $$d_{dc}=14.9947\times 10^{4}Z_{c}^{-0.46463}$$

SCS:

(15) $$d_{ds}=31.4148\times 10^{4}Z_{s}^{-0.46221}$$

## 4. Microstructure Evolution Simulation in Heavy Cylinder Rolling Process

### 4.1. Program Feasibility Verification

**Figure 6:**
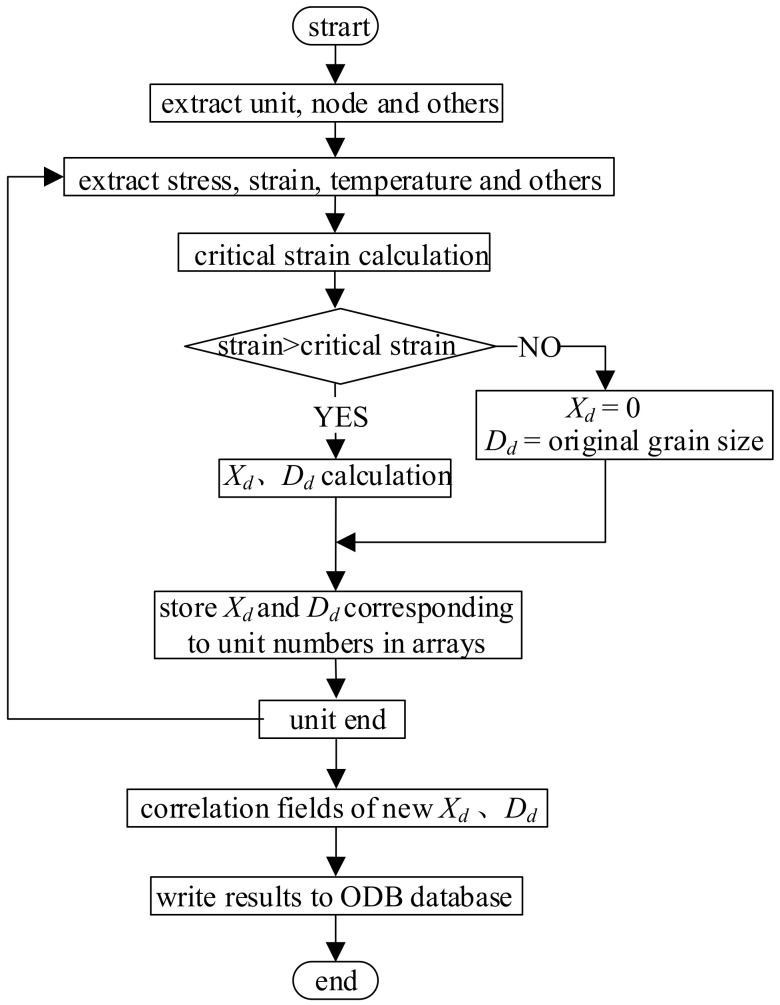
Program flow path.

It is feasible to simulate the evolution of a metal microstructure through a subroutine interface and script interface provided by the ABAQUS software, although this cannot be done directly. The mathematical model of dynamic recrystallization was defined as unit variables for an iterative operation through the second subroutine developed using the script interface of the ABAQUS software, through which the numerical simulation of the microstructure evolution of a heavy cylinder in the rolling process was realized. The general flow path of the program is shown in Figure 6, where *X_d_* and *D_d_* represent the dynamic recrystallization volume fraction and grain size, respectively.

**Figure 7:**
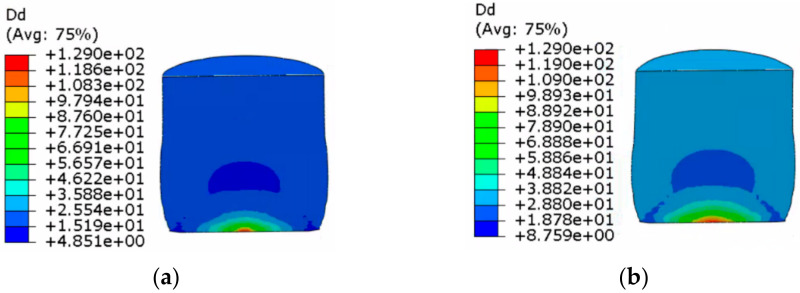
Grain size simulation of common cylindrical specimen: (**a**) T = 1050 °C, $\overset{\cdot }{\varepsilon }$ = 0.01 s^−1^ (**b**) T = 1150 °C, $\overset{\cdot }{\varepsilon }$ = 0.01 s^−1^.

First, some examples from the above compression experiments were selected to verify the feasibility of the program; the results are shown in Figure 7.

The following features can be seen in Figure 7:

(1) The grain distribution of the specimen was obviously divided and evenly distributed, and dynamic recrystallization occurred in most sections.
(2) The grain size at the bottom was larger than that in the other parts of specimen, and the grain size in the core was the smallest.
(3) The grain size and the range of the core region became larger with the increase in temperature.

Comparing the simulated grain size with the predicted grain size, as shown in Table 3, the results show that the error between them was small, indicating that the program could realize the simulation and prediction of microstructure evolution.

In conclusion, the feasibility of the development program was verified by the simulation of the grain size in the compression process.

### 4.2. Simulation of Microstructure Evolution in the Rolling Process of a Heavy Cylinder

**Figure 8:**
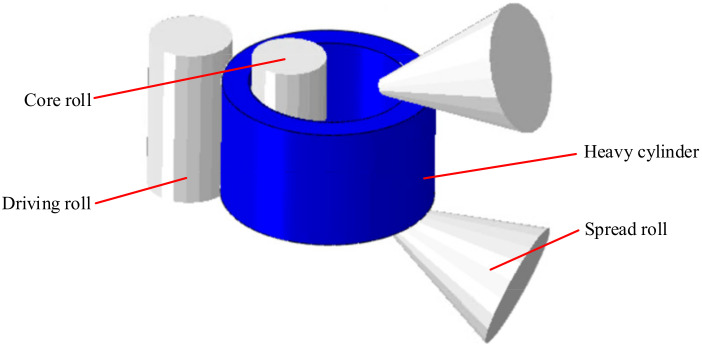
Finite element model of heavy cylinder.

The established finite element model for a heavy cylinder in the rolling process is shown in Figure 8. The rolling parameters were as follows: the outer diameter of the heavy cylinder was 5.1 m, the thickness was 0.625 m, the width was 3.0 m, the reduction in the first pass was 34 mm and the reduction in the second pass was 30 mm. In addition, a round pass was set after rolling. The model had the following characteristics: the driving roll and the core roll were set as rigid bodies and rotated around their own axes, the heavy cylinder was set as a rigid plastic body, and the deformation parameters in [19] were used. The friction between the rolls and heavy cylinder was Coulomb friction and the coefficient was 0.5. The initial temperature of the workpiece was defined as 1000 °C, and the roll and the ambient temperature were set to 30 °C. The contact heat transferred coefficient between the heavy cylinder and the roll was set to 10 N/(s mm °C), the thermal radiation coefficient was set to 0.8 N/(s mm °C), and the conversion coefficient of heat to work in plastic deformation was set to 0.9. In this work, the hexahedron unit was adopted to divide the net to calculate the simulation, and the total number of elements used for this model was 301,600. Mass scaling was performed to improve the calculation speed.

The dynamic recrystallization models of the SCS (considering shear effect) and common cylindrical specimen (without considering shear effect) established above were embedded into the rolling model of cylinder through the developed program. Thus, the microstructure changes during the rolling process were obtained. The following shows the results of the typical intercepted moments.

**Figure 9:**
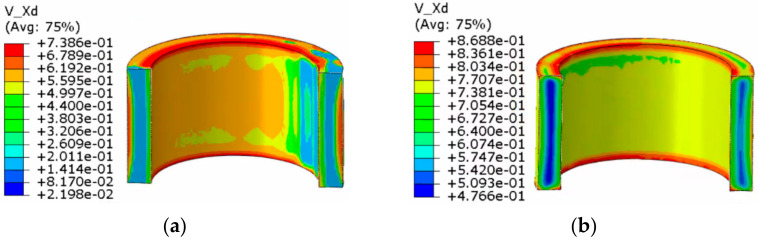
Nephogram of dynamic recrystallization volume fraction distribution without considering the shear effect: (**a**) first pass rolling; (**b**) second pass rolling.

**Figure 10:**
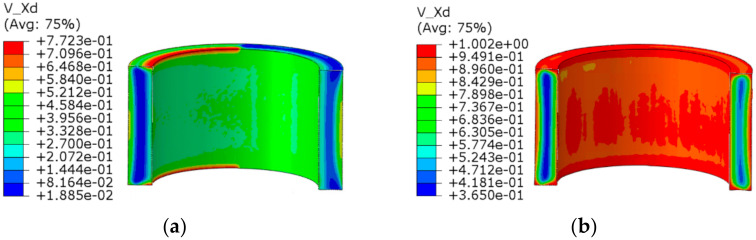
Nephogram of dynamic recrystallization volume fraction distribution considering the shear effect: (**a**) first pass rolling; (**b**) second pass rolling.

**Figure 11:**
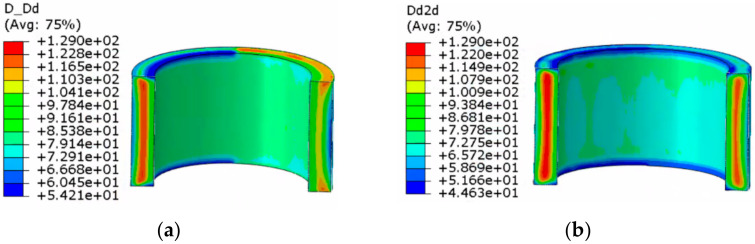
Nephogram of dynamic recrystallization grain size distribution without considering the shear effect: (**a**) first pass rolling; (**b**) second pass rolling.

**Figure 12:**
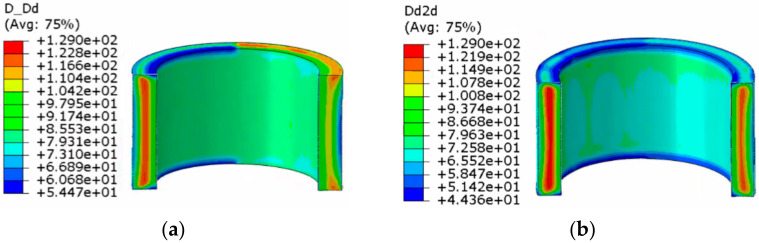
Nephogram of dynamic recrystallization grain size distribution considering the shear effect: (**a**) first pass rolling; (**b**) second pass rolling.

The dynamic recrystallization volume fraction distribution and grain size distribution nephograms without considering the shear effect and considering the shear effect are shown in Figure 9, Figure 10, Figure 11 and Figure 12.

The following can be seen in Figure 9 and Figure 10.

(1) In the whole rolling process, the recrystallization volume fraction was large and evenly distributed on the inside and outside of the cylinder due to direct contact with the roll, and the core of that was small.
(2) When shear effect was not considered, the dynamic recrystallization volume fraction reached 0.5323% at the end of first pass rolling. At the end of the second pass rolling, the dynamic recrystallization volume fraction reached 0.8190%. When the shear effect was considered, the dynamic recrystallization volume fraction reached 0.6764% at the end of first pass rolling and 0.9632% at the end of second pass rolling. This showed that a larger grain refinement range could be achieved by considering the shear effect in the rolling process of heavy cylinder.

The following can be seen in Figure 11 and Figure 12.

(1) In the whole rolling process, the recrystallization grain size was small and evenly distributed on the inside and outside of the cylinder due to direct contact with the roll, and the core was 129 μm, which was the initial grain size of the material. The grain size was smallest at the edge of the cylinder.
(2) When the shear effect was not considered, the dynamic recrystallization grain size reached 73.76 μm at the end of first pass rolling. At the end of the second pass rolling, the dynamic recrystallization grain size reached 70.26 μm. When the shear effect was considered, the dynamic recrystallization grain size reached 73.90 μm at the end of first pass rolling and 63.11 μm at the end of second pass rolling. This showed that the grain could be refined better when considering the shear effect.

In conclusion, the simulation results of the dynamic recrystallization microstructure evolution nephogram were obtained.

## 5. Analysis of Microstructure Evolution Characteristics in Heavy Cylinder Rolling Process

### 5.1. Path Setting of Microstructure Evolution

**Figure 13:**
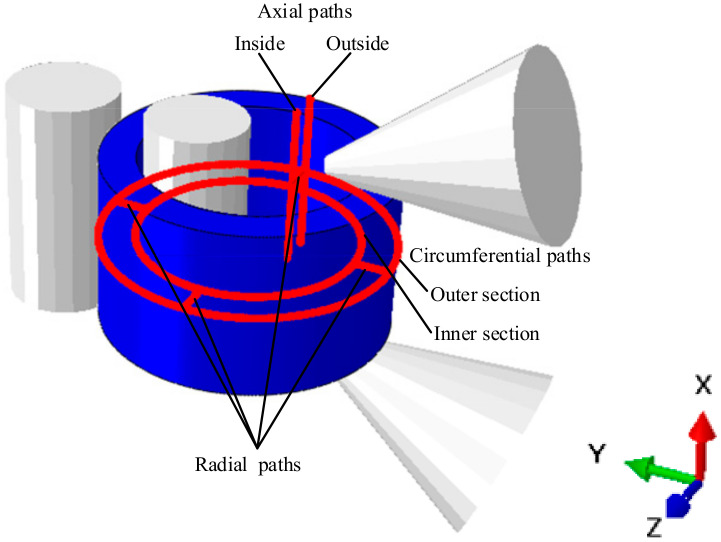
The axial, radial and circumferential paths.

In order to further analyze the results of the microstructure evolution during cylinder rolling, the characteristics of microstructure evolution were analyzed from axial, circumferential and radial paths. The path setting is shown in Figure 13, where the axial path includes the inside and outside, the circumferential path includes the inner section and outer section and the radial path direction is from the outer side to the inner side of the heavy cylinder.

### 5.2. Microstructure Evolution Characteristics of Axial Paths

**Figure 14:**
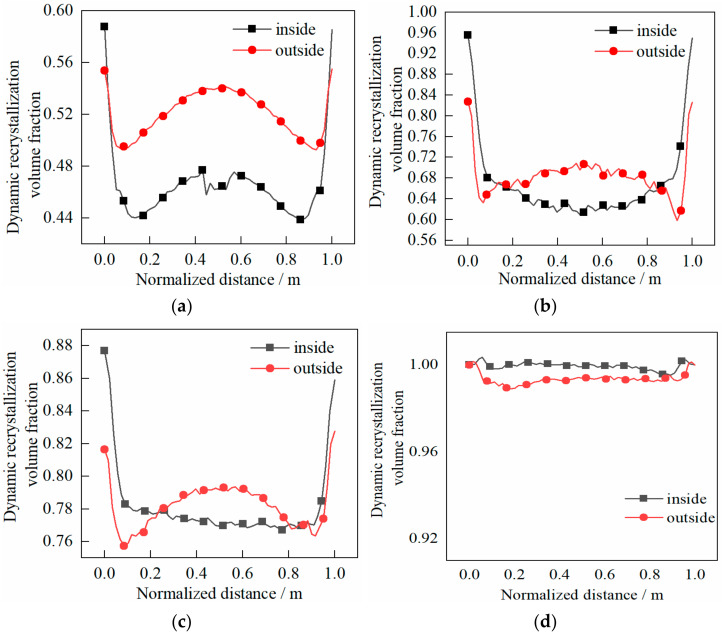
Evolution characteristics of recrystallization volume fraction in two passes of rolling: (**a**) first pass rolling, without shear effect; (**b**) first pass rolling, with shear effect; (**c**) second pass rolling, without shear effect; (**d**) second pass rolling, with shear effect.

**Figure 15:**
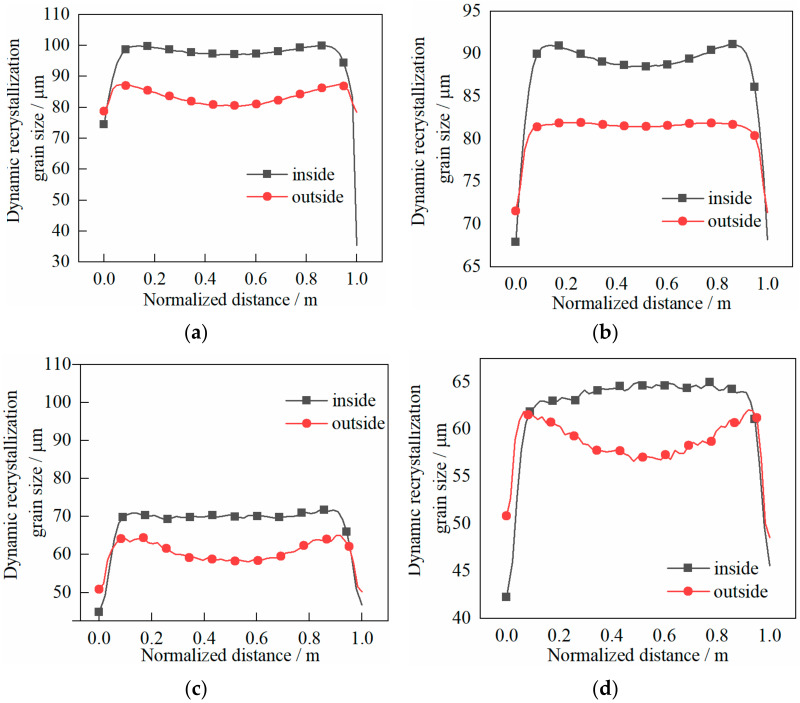
Evolution characteristics of recrystallization grain sizes in two passes of rolling: (**a**) first pass rolling, without shear effect; (**b**) first pass rolling, with shear effect; (**c**) second pass rolling, without shear effect; (**d**) second pass rolling, with shear effect.

The dynamic recrystallization volume fraction and grain size distribution characteristics of the cylinder are shown in Figure 14 and Figure 15, respectively.

The following can be seen in Figure 14.

(1) The volume fraction on the outside of the cylinder was larger than that on the inside, which indicated that the outside was more prone to dynamic recrystallization.
(2) At the end of first pass, the volume fraction in the core was larger than that at the two ends when the shear effect was not considered. The volume fraction difference between the inside and outside was about 0.07%, while the volume fraction in the core showed few differences with the two ends when the shear effect was considered. The volume fraction difference between the inside and outside was about 0.02%.
(3) At the end of the second pass, the difference of the volume fraction between the core and the two ends decreased when the shear effect was not considered. The volume fraction difference between the inside and outside was less than 0.02%, and the volume fraction in the core was roughly the same as that at the two ends when the shear effect was considered. The volume fraction difference between the inside and outside was about 0.01%.

The following can be seen in Figure 15.

(1) The grain size on the outside was smaller than that on the inside, which indicated that the grain on the outside was refined better.
(2) At the end of first pass rolling, the grain size in the core was roughly the same as that at both ends when the shear effect was not considered. The grain size difference between the inside and outside was about 15 μm, while the grain size in the core was basically the same as that in the both ends when the shear effect was considered. The difference between the grain size inside and outside was no more than 10 μm.
(3) At the end of the second pass, the grain size difference between the inside and outside was about 10 μm when the shear effect was not considered, and the grain size difference was no more than 8 μm when the shear effect was considered.

### 5.3. Microstructure Evolution Characteristics of Circumferential Paths

**Figure 16:**
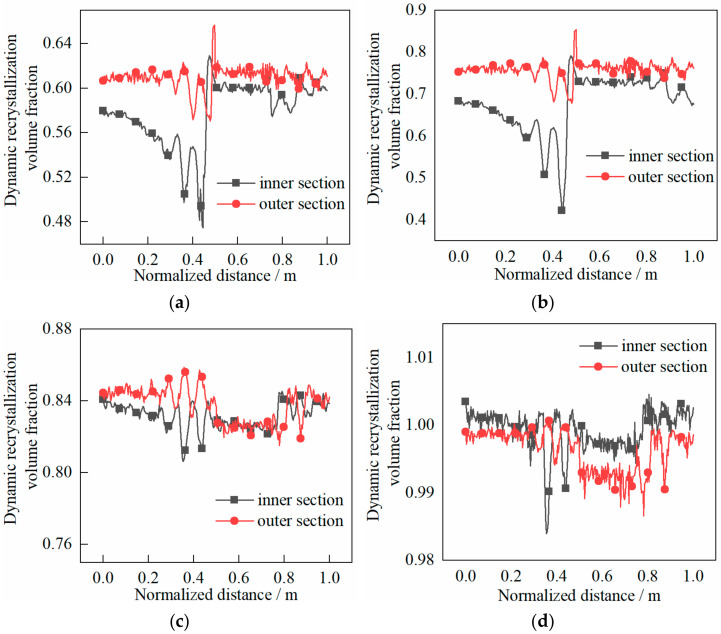
Evolution characteristics of recrystallization volume fraction in two passes of rolling: (**a**) first pass rolling, without shear effect; (**b**) first pass rolling, with shear effect; (**c**) second pass rolling, without shear effect; (**d**) second pass rolling, with shear effect.

**Figure 17:**
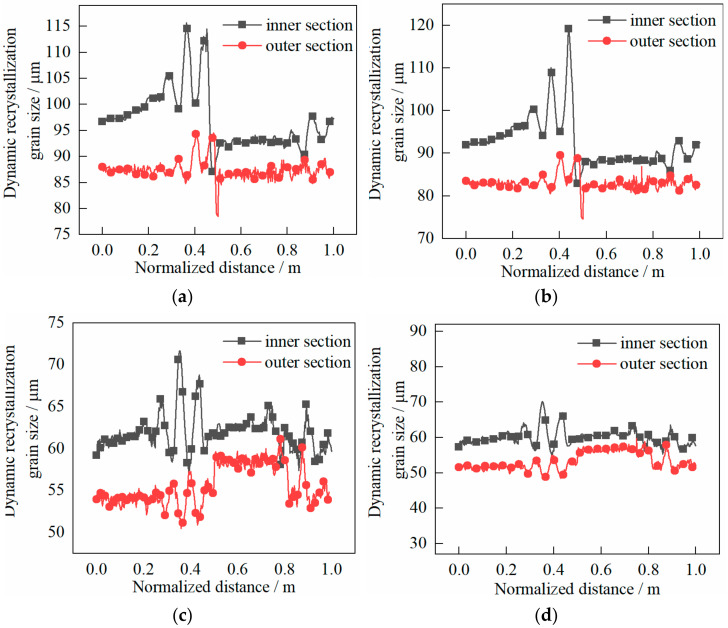
Evolution characteristics of recrystallization grain size in two passes of rolling: (**a**) first pass rolling, without shear effect; (**b**) first pass rolling, with shear effect; (**c**) second pass rolling, without shear effect; (**d**) second pass rolling, with shear effect.

The dynamic recrystallization volume fraction and grain size distribution characteristics of the cylinder are shown in Figure 16 and Figure 17, respectively. 

The following can be seen in Figure 16.

(1) In the rolling process, the volume fraction on the outer section of the cylinder was larger than that on the inner section and more evenly distributed. The abrupt change of volume fraction was due to the biting in and out of the heavy cylinder.
(2) At the end of the first pass, the volume fraction was about 0.60% when the shear effect was not considered, while the volume fraction was about 0.75% when the shear effect was considered;
(3) At the end of the second pass, the volume fraction was about 0.84% when shear effect was not considered, while the volume fraction was about 1.00% when the shear effect was considered.

The following can be seen in Figure 17.

(1) The grain size on the outer section was smaller than that on the inner section and more evenly distributed. The abrupt change of grain size was due to the biting in and out of the heavy cylinder.
(2) At the end of the first pass, the grain size was about 90 μm when the shear effect was not considered, while the grain size was about 85 μm when the shear effect was considered;
(3) At the end of the second pass, the grain size was about 58 μm when the shear effect was not considered, while the grain size was about 55 μm when the shear effect was considered.

### 5.4. Microstructure Evolution Characteristics of Radial Paths

**Figure 18:**
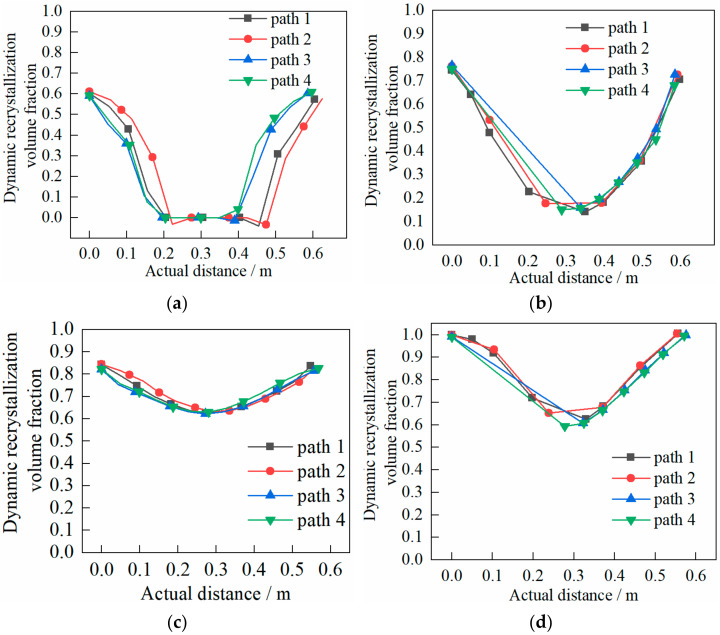
Evolution characteristics of recrystallization volume fraction in two passes of rolling: (**a**) first pass rolling, without shear effect; (**b**) first pass rolling, with shear effect; (**c**) second pass rolling, without shear effect; (**d**) second pass rolling, with shear effect.

**Figure 19:**
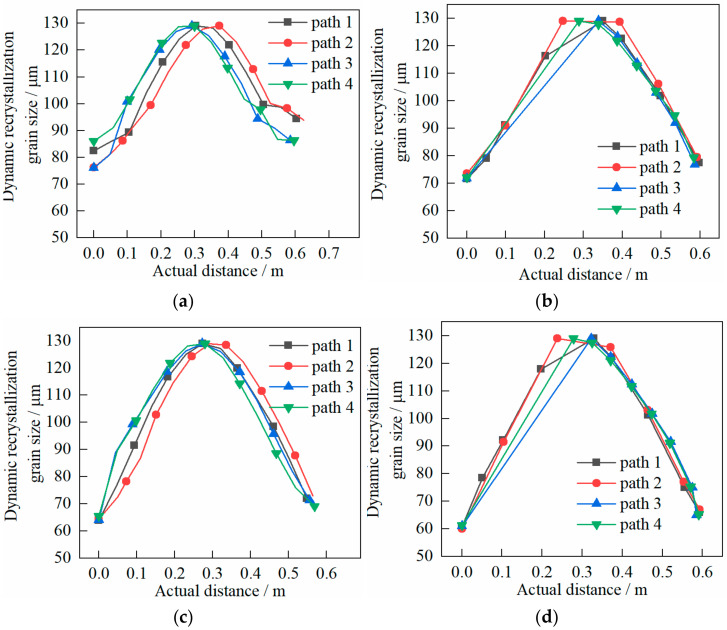
Evolution characteristics of recrystallization grain size in two passes of rolling: (**a**) first pass rolling, without shear effect; (**b**) first pass rolling, with shear effect; (**c**) second pass rolling, without shear effect; (**d**) second pass rolling, with shear effect.

The dynamic recrystallization volume fraction and grain size distribution characteristics of the cylinder are shown in Figure 18 and Figure 19, respectively. 

The following can be seen in Figure 18.

(1) The volume fraction inside and outside of the cylinder was larger than that in the core. The change from inside and outside to the core was faster.
(2) At the end of the first pass, the volume fraction was about 0.60% when the shear effect was not considered. While there was no dynamic recrystallization in the core, significant dynamic recrystallization occurred in the inside and outside. The volume fraction was about 0.75% when the shear effect was considered. However, there was slight dynamic recrystallization in the core, and significant dynamic recrystallization occurred in the inside and outside. This result was consistent with that of circumferential evolution.
(3) At the end of the second pass, the volume fraction was about 0.85% and that of the core was about 0.62% when the shear effect was not considered, while dynamic recrystallization occurred over the whole heavy cylinder. The volume fraction was about 1.00% and that of the core was about 0.60% when the shear effect was not considered, and dynamic recrystallization occurred over the whole heavy cylinder. Again, this result was consistent with that of circumferential evolution.

The following can be seen in Figure 19.

(1) The grain size inside and outside of the cylinder was smaller than that in the core. The change from inside and outside to the core was faster.
(2) At the end of the first pass, the grain size of inside and outside was about 90 μm while the core showed the original grain size of 129 μm when the shear effect was not considered. The grain size inside and outside was about 80 μm and the core was the original grain size of 129 μm when the shear effect was considered. This result was roughly consistent with that of circumferential evolution.
(3) At the end of the second pass, the grain size inside and outside was about 66 μm and the core was the original grain size of 129 μm when the shear effect was not considered. The grain size inside and outside was about 61 μm and the core was the original grain size of 129 μm when the shear effect was considered. This result was not very different from that of circumferential evolution.

In conclusion, when considering the shear effect, dynamic recrystallization was more likely to occur in the rolling process. Meanwhile, the grain size was smaller and the microstructure was refined better.

## 6. Conclusions

In this paper, an SCS and common cylindrical specimen were designed to study the shear effect on microstructural evolution under different deformation conditions, and a corresponding dynamic recrystallization kinetic model and grain size model were established. The results showed that the dynamic recrystallization deformation of SCS occurs more easily, and the grain size is better refined under the same deformation conditions. A secondary development program of microstructure evolution simulation was compiled based on the finite element software and coupled with the finite element software. The program was first verified by the physical simulation of the common cylindrical specimen. Then, the program was coupled with the rolling process of a heavy cylinder to establish a macro-micro coupled model to study the microstructure evolution in the cylinder rolling process. The microstructure evolution characteristics of the rolling process were further analyzed from the axial, circumferential and radial directions, respectively. The results showed that the dynamic recrystallization of the cylinder occurred more easily when considering the shear effect, and the grain refinement result was more obvious. Compared with the values without considering the shear effect, the volume fraction of dynamic recrystallization increased by 0.25%, and the grain size was refined by 30 μm.

## Figures and Tables

**Table 1:** Chemical composition of 2.25Cr1Mo0.25V steel (wt %).

C	Si	Cr	Mn	P	Mo	V	Ti	B
0.150	0.040	2.250	0.58	0.005	1.000	0.027	0.020	0.001

**Table 2:** Partial prediction data of dynamic recrystallization grain size.

Category	Deformation Temperature *T* (°C)	Strain Rate (s^−1^)	Grain Size *d_d_* (μm)
Common cylindrical specimen	1050	0.001	35
	1050	0.01	12
	1150	0.001	91
	1150	0.01	34
SCS	1050	0.001	25
	1050	0.01	8
	1150	0.001	70
	1150	0.01	24

**Table 3:** Average grain size error of some specimens.

Figure	Simulation Value (μm)	Predicted Value (μm)	Relative Error (%)
Figure 6a	13.05	12	8.75
Figure 6b	32.12	34	5.53

## Data Availability

Data available on request due to restrictions e.g., privacy.
